# Barriers and Facilitators to Early Postpartum Blood Pressure Follow‐Up After Hypertensive Disorders of Pregnancy: An Integrative Review

**DOI:** 10.1111/jmwh.70091

**Published:** 2026-03-26

**Authors:** Jill C. Doyle, Christopher S. Lee, Lucinda Canty

**Affiliations:** ^1^ William F. Connell School of Nursing Boston College Chestnut Hill Massachusetts; ^2^ Elaine Marieb College of Nursing University of Massachusetts Amherst Amherst Massachusetts

**Keywords:** blood pressure monitoring, health equity, hypertensive disorders of pregnancy, intersectionality, maternal health disparities, postpartum care, preeclampsia

## Abstract

**Introduction:**

Hypertensive disorders of pregnancy (HDP) are a leading cause of preventable maternal morbidity and mortality in the United States. Because blood pressure (BP) often peaks after hospital discharge, clinical guidelines recommend BP evaluation within 7 to 10 days postpartum. Yet fewer than half of postpartum individuals with HDP receive this follow‐up, with the lowest rates among Black and Hispanic individuals. This integrative review aimed to identify multilevel barriers, facilitators, and predictors of timely postpartum BP follow‐up using an intersectionality framework to inform equity‐focused care.

**Methods:**

PubMed, CINAHL, Scopus, and Web of Science were searched in February 2025 for studies published between May 2018 and February 2025. Eligible studies included quantitative, qualitative, or mixed‐methods research that examined BP evaluation within 10 days postpartum among individuals with HDP; case reports and studies without racial/ethnic data were excluded. Thirteen studies met the criteria. Data were extracted into an evidence table, appraised with the Johns Hopkins Evidence‐Based Practice model, and analyzed to identify multilevel barriers, facilitators, and predictors of follow‐up.

**Results:**

Barriers to follow‐up included racial and ethnic inequities, inadequate insurance, socioeconomic disadvantage, and fragmented prenatal care. Facilitators included remote BP monitoring, nurse‐led interventions, and supportive provider communication. Predictors such as HDP severity, cesarean birth, maternal age, and parity were associated with follow‐up, reflecting structural and contextual influences on postpartum care engagement.

**Discussion:**

Disparities in early postpartum BP follow‐up are rooted in intersecting systems of racism, classism, and structural disadvantage. These findings align with prior research showing the benefits of remote BP monitoring but extend the literature by identifying populations that remain excluded. Equity‐focused strategies, including remote BP monitoring, midwifery‐led care, and policy reform, are urgently needed to improve postpartum outcomes and reduce disparities. Further research should evaluate how these strategies can be implemented to address structural barriers and engage populations most affected by HDP.

## INTRODUCTION

Hypertensive disorders of pregnancy (HDP), including preeclampsia, gestational hypertension, and chronic hypertension, affect 13% to 15% of pregnancies in the United States^1^ and are a leading cause of preventable postpartum morbidity and mortality.^2^ Most maternal deaths and a substantial proportion of severe morbidity related to HDP occur in the early postpartum period.^3^, ^4^ During this time, blood pressure (BP) typically peaks between postpartum days 3 and 7, increasing the risk of complications, such as eclamptic seizure, stroke, and cardiomyopathy.^4^, ^5^, ^6^, ^7^, ^8^ In recognition of this high‐risk period, the American College of Obstetricians and Gynecologists (ACOG) recommends a BP evaluation within 7 to 10 days postpartum for individuals with HDP.^9^ International guidelines echo the importance of early postpartum monitoring. The World Health Organization recommends a minimum of 4 postnatal care contacts for all individuals who are postpartum, including between 7 and 14 days after birth, to enable timely detection and management of postpartum complications—such as hypertensive disorders—and to ensure equitable access to high‐quality care, particularly for those from marginalized populations.^10^ Early postpartum surveillance (eg, BP evaluation within 7‐10 days after birth) is recommended for all individuals with HDP, regardless of provider type.^9^, ^11^ The American Heart Association (AHA) emphasizes multidisciplinary care, noting that nurses and midwives play key roles in postpartum BP surveillance, care coordination, and early recognition of maternal compromise.^12^ Collaborative care models that include midwives in postpartum follow‐up may help close care gaps, improve health care equity, and facilitate education about long‐term cardiovascular risk after an HDP.^12^ Although direct evidence comparing provider types remains limited, current recommendations prioritize timely monitoring and coordination over provider restrictions.

1 **QUICK POINTS**

✦ Early postpartum blood pressure follow‐up after hypertensive disorders of pregnancy (HDP) is low overall, with the lowest rates among patients who are Black and Hispanic, who also face the highest risk of severe complications.
✦ Barriers to follow‐up include structural racism, inadequate insurance, limited care access, and digital exclusion; facilitators include remote blood pressure monitoring, nurse‐led education, and supportive provider communication.
✦ Younger maternal age, multiparity, and less severe HDP are associated with lower follow‐up rates and may identify patients needing additional outreach.
✦ Relationship‐centered care models that address social determinants and structural barriers and expand equitable access to remote monitoring and community supports are essential to reduce disparities.

Despite these clinical guidelines, fewer than 50% of postpartum persons with HDP receive a BP evaluation within the recommended 7 to 10 days postpartum.^13^, ^14^, ^15^ Follow‐up rates are especially low among Black and Hispanic postpartum persons, who also face higher risks of both developing HDP and experiencing more severe complications.^16^, ^17^, ^18^, ^19^ Black women, in particular, have a 2‐fold higher risk of stroke and severe morbidity and are significantly less likely to receive timely postpartum BP monitoring.^11^, ^20^ National surveillance data show that Black women are 5 times more likely to die from HDP‐related causes than White women.^11^, ^21^ These disparities stem not from clinical risk alone but from structural racism, unequal access to quality care, and broader social determinants of health.^22^, ^23^, ^24^ Together, these inequities highlight the importance of timely postpartum surveillance and the risks of missed follow‐up in the early weeks after birth.

The urgency of early follow‐up is further underscored by the timing of postpartum stroke, the leading cause of HDP‐related mortality, which most often occurs in the first 10 days after birth, coinciding with the period when BP levels peak.^4^, ^5^ Because this high‐risk window begins after hospital discharge, follow‐up depends on outpatient access during a time when patients are physically and emotionally vulnerable. Many patients who are postpartum—particularly women of color—encounter structural, logistical, and psychosocial barriers that impede timely evaluation.^23^, ^25^, ^26^ Despite its clinical importance, the mechanisms driving these inequities remain insufficiently understood.^24^ In response, innovative models, such as remote BP monitoring (RBPM), have been introduced to expand access and reduce gaps in early follow‐up.

To address these barriers, RBPM has emerged as a promising strategy to improve postpartum BP follow‐up beyond traditional office visits.^13^, ^14^, ^27^, ^28^ RBPM involves using home BP devices to measure and electronically transmit readings to clinicians, who review values and provide management guidance.^29^ This differs from traditional home BP monitoring (HBPM), in which patients measure BP at home but manually report readings during follow‐up. According to the American College of Cardiology and the AHA, both RBPM and HBPM can provide accurate and reliable out‐of‐office BP measurements when validated devices and proper technique are used, but RBPM offers additional advantages by enabling timely clinician review and intervention.^30^ Although there is evidence that RBPM improves postpartum BP ascertainment and may reduce racial disparities in follow‐up,^29^, ^31^ few studies have examined inequities in digital access, patient engagement, and technological infrastructure.^32^, ^33^ Without equity‐centered implementation, RBPM could inadvertently reproduce disparities.^34^ This underscores the need to move beyond single interventions and to understand the broader multilevel factors that shape early postpartum BP follow‐up.

To date, limited research has comprehensively examined these factors at the patient, provider, and system levels and how they influence early postpartum BP follow‐up. This integrative review addresses that gap by identifying the barriers, facilitators, and predictors of the 7‐to‐10‐day postpartum BP evaluation. Guided by an intersectionality framework,^35^, ^36^ this review explores how overlapping social identities and structural determinants—such as race, socioeconomic status, and health care access—interact to shape postpartum care. By identifying actionable targets for intervention, this work aims to inform equity‐focused models of care and reduce preventable maternal morbidity and mortality.

### Theoretical Framework

This review is guided by intersectionality theory, a framework rooted in Black feminist thought and legal scholarship that examines how overlapping social identities, such as race, gender, and socioeconomic status, interact with systems of oppression.^36^, ^37^, ^38^ Intersectionality highlights how structures such as racism, classism, and sexism compound to produce health care inequities not explained by individual risk factors alone,^35^ and it is increasingly recognized in public health research as essential for designing equity‐focused interventions.^39^, ^40^ Because HDP‐related complications disproportionately affect Black and Hispanic individuals, this review applies an intersectional lens to examine multilevel contributors to timely postpartum BP follow‐up in these populations. Using this framework allows for a nuanced analysis of how patient‐, provider‐, and system‐level factors converge to shape postpartum care experiences.

While acknowledging that not all pregnancy‐capable individuals identify as women, this review uses the terms “woman” and “women” to reflect the language used in the included studies. When possible, gender‐inclusive language, such as “postpartum persons,” is used to honor the diversity of birthing experiences and identities.

## METHODS

An integrative review methodology was used to synthesize evidence from both quantitative and qualitative studies. This approach was selected to reflect the complexity of factors influencing postpartum BP evaluation and to integrate findings across diverse research designs. It is particularly well suited to this topic because it accommodates clinical outcomes, patient experiences, and structural barriers relevant to equitable postpartum care. The review followed Whittemore and Knafl's 5‐stage process: 1) problem identification, 2) literature search, 3) data evaluation, 4) data analysis, and 5) presentation of findings.^41^ To enhance transparency in the literature search and study selection, this review is reported in accordance with the Preferred Reporting Items for Systematic Reviews and Meta‐Analyses (PRISMA) 2020 guidelines^42^ (Supporting Information: Appendix S1).

A preliminary search of ProQuest, the Cochrane Library, and PROSPERO confirmed that no existing reviews addressed this topic. The review question was structured using the Population, Concept, Context framework,^43^ which guided inclusion criteria and search strategy. In this framework, the population was defined as women of color with HDP, the concept was timely postpartum BP evaluation, and the context involved factors affecting racially and ethnically minoritized populations in the United States.

A systematic search was conducted in February 2025 across 4 electronic databases: PubMed, CINAHL, Web of Science, and Scopus. Search terms reflected 3 primary constructs—postpartum, BP evaluation, and HDP—and were tailored for each database using Medical Subject Headings, keywords, synonyms, truncation, and Boolean operators. Filters were applied to limit results to full‐text, peer‐reviewed articles published in English between May 2018 and February 2025 involving human participants. This time frame was selected to capture literature published after ACOG issued its 2018 Committee Opinion number 736: *Optimizing Postpartum Care*, which formally recommended BP evaluation within 7 to 10 days postpartum for individuals with HDP.^9^

Studies were included if they met the following criteria: 1) examined BP evaluation within 10 days postpartum; 2) focused on patient‐, provider‐, or system‐level factors influencing BP follow‐up; 3) employed a quantitative, qualitative, mixed‐methods, or systematic review design; 4) were conducted in the United States; and 5) sampled women of color diagnosed with HDP, including preeclampsia, gestational hypertension, chronic hypertension, or preeclampsia superimposed on chronic hypertension. Studies were excluded if they did not focus on the postpartum period, did not address BP evaluation, or were not original research. Case reports, abstracts, opinion pieces, dissertations, and guidelines were also excluded.

**Figure 1:**
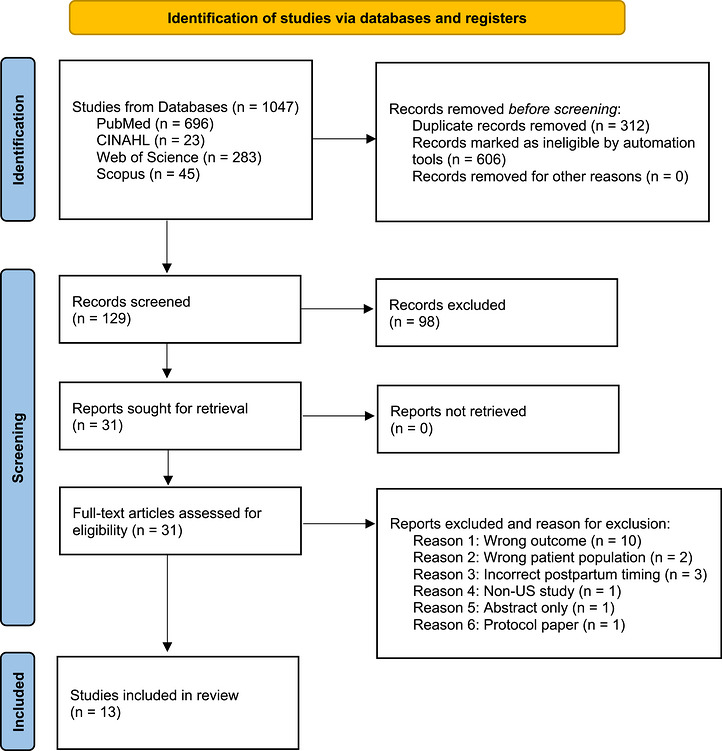
Preferred Reporting Items for Systematic Reviews and Meta‐Analyses (PRISMA) Flow Diagram of the Study Selection Process

Search results were imported into Zotero reference management software for deduplication, organization, and full‐text review. A single reviewer (first author) independently screened all title and abstracts against the eligibility criteria, followed by full‐text review of potentially eligible studies. The screening and selection process is illustrated in the PRISMA flow diagram (Figure 1).

A total of 1047 records were retrieved. After removing 312 duplicates, 735 unique records remained. Of these, 606 were excluded during initial screening for not meeting eligibility criteria, leaving 129 articles for title and abstract review. Ninety‐eight articles were excluded at this stage. Full‐text review was conducted for the remaining 31 articles. Following full‐text review, 18 articles were excluded for the following reasons: 1) outcome not focused on postpartum BP (n = 10), 2) population not meeting inclusion criteria (n = 2), 3) incorrect postpartum timing of BP evaluation (n = 3), 4) non‐US setting (n = 1), 5) abstract only (n = 1), and 6) guideline article (n = 1). Thirteen studies met all inclusion criteria and were retained for analysis.

The level and quality of evidence of each included study were assessed using the Johns Hopkins Evidence‐Based Practice (JHNEBP) model,^44^ which classifies studies by level of evidence (I‐V) based on study design and quality rating (A‐C) based on methodological rigor, consistency, and generalizability of results. All studies were rated using this model, and none were excluded based on quality.

Data extraction and analysis followed Whittemore and Knafl's integrative review methodology.^41^ The first author independently extracted data from all included studies into a standardized evidence table (Table 1), capturing study authors, year of publication, aim, design, design and methods, sample and location, reported race/ethnicity, language inclusion, main findings, and evidence level and quality (JHNEBP appraisal). Studies were grouped by research design and thematically analyzed to identify patient‐level, provider‐level, and system‐level factors influencing early postpartum BP follow‐up, further categorized as barriers, facilitators, or predictors.

**Table 1:** Summary of Included Studies on Early PP BP Follow‐Up After HDP

Author (Year)	Study Aim	Design and Methods	Sample and Setting	Race/Ethnicity (%)^a^	Language Inclusion	Main Findings	Level of Evidence and Quality^b^
Arkerson et al (2023)^27^	Compare the rate of BP ascertainment within 10 days of PP discharge among individuals with HDP randomized to in‐office BP assessment or at‐home monitoring	Multisite randomized controlled trial	197 PP patients with HDP before discharge at 2 US academic medical centers (locations not reported)	Black (32), White (58), Hispanic (8), Asian (2)	English only^c^	BP ascertainment within 10 days was significantly higher with RBPM compared with office‐based care (92% vs 58%). Among Black patients, RBPM eliminated the racial disparity in BP follow‐up seen with in‐office surveillance (93% vs 41%). Patient satisfaction with RBPM monitoring was high.	Level I‐A
Campbell et al (2022)^15^	Describe predictors of PP BP screening attendance	Population‐based retrospective cohort study	1260 PP patients with HDP at Grady Memorial Hospital in Atlanta, Georgia	Black (81), White (3), Hispanic (14), other or unknown (2)	Not specified	Only 13.7% attended a 10‐day BP check. Severe preeclampsia and adequate prenatal care were associated with higher attendance. Although not statistically significant, lower attendance was observed among individuals with non‐Hispanic Black race, public insurance, and high parity.	Level III‐B
Howard et al (2024)^45^	Evaluate whether Black and White patients enrolled in an online monitoring program showed improvements in BP ascertainment	Retrospective cohort study	3976 PP patients with HDP at a hospital system in Louisiana	Black (37), White (63), Hispanic (4)	Not specified	Black patients had lower enrollment in the online BP monitoring program than those who were White. The intervention improved 7‐day BP ascertainment for patients who were White but not for patients who were Black.	Level III‐B
Janssen et al (2021)^46^	Measure rate of text‐based BP monitoring program use during the first 10 days after hospital discharge	Prospective observational cohort study	199 PP patients with HDP at 3 US academic medical centers (locations not reported)	Black (34), White (42), Hispanic (12), Asian (2), other or unknown (11)	English only^c^	More patients enrolled in the text‐based BP monitoring program submitted a BP measurement on PP days 7‐10 than on days 1‐4.	Level III‐B
Lemon et al (2024)^28^	Evaluate differences in health care use and guideline adherence for individuals who are PP with HDP who are engaged in a RBPM program, compared with usual care	Retrospective cohort study	12,038 PP patients with HDP (6556 in RBPM program vs 5482 in control group) at the University of Pittsburgh School of Medicine Magee‐Womens Hospital (Pittsburgh, PA)	Black (22), White (71), Hispanic (2), other or unknown (7)	English, Spanish, or Portuguese	Participants with RBPM were more likely to be White, commercially insured, and diagnosed with preeclampsia. RBPM was associated with higher PP visit attendance and better guideline adherence.	Level III‐B
Moustafa et al (2024)^47^	Determine whether patient education and home BP telemonitoring increased PP BP monitoring and PP visit attendance	Prospective cohort study	250 PP patients with HDP at the University of Mississippi Medical Center (Jackson, MS)	Black (74), White (17), Hispanic (3), other or unknown (2)	English or Spanish	Among participants of the study, 39% attended the 10‐day PP BP check visit. Standardized education and RBPM were not associated with improved follow‐up. Patients who were younger, Black, and Hispanic demonstrated lower engagement in the RBPM program, even after adjusting for community‐level distress.	Level III‐B
Patel et al (2024)^48^	Describe PP visit attendance and PP BP control among patients enrolled in RBPM program and compare outcomes by race	Prospective cohort study	545 PP patients with HDP at the University of Chicago (Chicago, IL)	Black (65), White (18), Hispanic (12), Asian (5), multiracial (7)	Not specified	Attendance at the 7‐ to 10‐day PP BP check improved from 76% (telehealth only) to 84% with the RBPM program. Among participants with RBPM, no significant racial disparity in BP check attendance was observed. Public insurance was the only factor independently associated with lower attendance. Over time, engagement declined, with Black patients more likely to become nonresponders; these patients were also younger, publicly insured, and less likely to have received prenatal care at the study hospital.	Level III‐B
Payakachat et al (2020)^53^	Explore perceptions and attitudes of PP women with preeclampsia toward RBPM and communication with the call center	Qualitative cohort study, phone interviews	37 PP patients with preeclampsia (21 users of RBPM and 16 nonusers) at the University Medical Center (Little Rock, AR)	Black (50), White (50)	English only^c^	Both users and nonusers of the RBPM program perceived it as helpful. Users valued daily monitoring and nurse communication; barriers included equipment fit, wireless issues, and stress. Nonusers cited difficulty integrating the program into their routine.	Level III‐C
Romagano et al (2020)^49^	Identify factors associated with patient attendance at the PP BP follow‐up visit	Retrospective cohort study	378 PP patients with HDP at University Hospital (Newark, NJ)	Black (60), Hispanic (35), other or unknown (5)	Not specified	Attendance at the BP check visit was higher among patients with preeclampsia or cesarean birth. Black non‐Hispanic race and gestational hypertension were associated with lower follow‐up rates, with race remaining a significant factor in adjusted models.	Level III‐B
Sanghavi et al (2022)^50^	Compare completion rate of new patient telemedicine visits to in‐person office visits for patients with preeclampsia	Retrospective cohort study	236 PP patients with HDP at the Hospital at the University of Pennsylvania (Philadelphia, PA)	Black (70), Hispanic (3), race not reported (27)	Not specified	PP BP visit completion was significantly higher with telemedicine (70%) than in‐person visits (32%). Patients who were Black and younger were less likely to complete in‐person visits, but this disparity was not observed with telemedicine. Show rates for both visit types did not differ by SES or disease severity.	Level III‐B
Suresh et al (2021)^51^	Test ability of hospital QI initiative to improve PP BP control and completion of PP follow‐up among patients with HDP	QI initiative	926 PP patients with HDP at the University of Chicago (Chicago, IL)	Black (80), White (13), Hispanic (5), Asian (2), Native Hawaiian/Pacific Islander (0.1), multiracial (3), other or Unknown (2)	Not specified	PP BP visit attendance increased from 34% to 59% after a nurse‐led QI bundle—particularly after hiring a nurse educator. However, patients who were younger, Black, and publicly insured remained less likely to attend.	Level V‐B
Tallmadge et al (2021)^52^	Assess maternal characteristics that predict attendance of PP BP evaluation in patients with HDP	Retrospective case‐control study	1041 PP patients with HDP at Froedtert and the Medical College of Wisconsin (Milwaukee, WI)	Black (40), White (50), other or unknown (10)	Not specified	Factors associated with higher odds of attending the PP BP check visit included nulliparity, severe HDP, and cesarean birth. Lower odds of attendance were associated with non‐Hispanic Black race/ethnicity compared with non‐Hispanic White and public insurance compared with private insurance.	Level III‐B
Tully et al (2024)^54^	Compare PP BP documentation via RBPM with text message reminders vs office‐based follow‐up and explore barriers and facilitators of both care strategies from patient perspectives	Single‐site randomized controlled trial; mixed methods	100 PP patients (50 per arm) with HDP at North Carolina Women's Hospital (Chapel Hill, NC)	Black (28), White (45), Hispanic (23), Asian (3)	English or Spanish	PP BP evaluation was higher with RBPM and text reminders (76%) vs standard care (58%), though not statistically significant. Overall follow‐up improved from 20% to 67%. Participants found RBPM acceptable, citing convenience, clear instructions, and reassurance as facilitators; barriers included newborn demands and limited access to PP health information.	Level I‐B

Abbreviations: BP, blood pressure; HDP, hypertensive disorders of pregnancy; PP, postpartum; QI, quality improvement; RBPM, remote BP monitoring; SES, socioeconomic status.

a Percentages are approximate and based on demographic data reported in each study. Calculations were made using the available sample sizes and may not fully capture the complexity of racial and ethnic identity or the categorization of Hispanic ethnicity.

b Levels of evidence (I‐V) and quality (A‐C) from the Johns Hopkins Evidence‐Based Practice model rating scale.^44^

c These studies explicitly excluded participants who do not speak English.

### Reflexivity

The first author (J.C.D.) identifies as a White, cisgender, socioeconomically privileged woman, nurse‐midwife, doctoral student, and mother with lived experience of postpartum preeclampsia. These intersecting personal and professional identities influenced how the evidence was interpreted and which themes were emphasized. Guided by the intersectionality framework, reflexivity was central to the analytic process. Critical self‐reflection, peer debriefing, and co‐author feedback enhanced transparency, minimized bias, and strengthened the rigor and equity orientation of this review.

## RESULTS

### Study Characteristics

Thirteen studies met the inclusion criteria: 11 quantitative,^15^, ^27^, ^28^, ^45^, ^46^, ^47^, ^48^, ^49^, ^50^, ^51^, ^52^ one qualitative,^53^ and one mixed‐methods study with a randomized controlled trial (RCT) component.^54^ Among the quantitative studies, 2 were RCTs,^27^, ^54^ 3 were prospective cohorts,^46^, ^47^, ^48^ 6 were retrospective cohorts,^15^, ^28^, ^45^, ^49^, ^50^, ^52^ and one was a quality improvement (QI) initiative.^51^

Most studies were rated as level III (nonexperimental) and of good quality (B) based on the JHNEBP model. The qualitative study by Payakachat et al,^53^ was rated level III‐C because of poorly defined methodology and limited generalizability. The 2 RCTs provided higher‐level evidence: Arkerson et al^27^ was rated level I‐A for strong design and rigor, and Tully et al,^54^ a mixed‐methods study with an RCT component, was rated level I‐B because of minor methodological limitations. Overall, the evidence base was of good quality, with most studies rated level III‐B and only 2 RCTs providing higher‐level evidence.

Geographically, 5 studies were conducted in the South,^15^, ^45^, ^47^, ^53^, ^54^ 3 were conducted in the Midwest,^48^, ^51^, ^52^ and 3 were conducted in the Northeast.^28^, ^49^, ^50^ Two studies did not specify geographic region.^27^, ^46^ Together, these studies reflect a range of regions across the United States, but variation in participant demographics and clinical characteristics provides further context for interpreting their findings.

### Participant Demographics and Clinical Characteristics

Across these studies, participants were women who were postpartum and diagnosed with HDP. Sample sizes ranged from 37 to 12,038 participants, with mean ages between 26 and 31 years, consistent with national maternal age trends.^55^

Racial and ethnic diversity was represented across all studies. All 13 studies included Black participants, and 11 also included White participants.^15^, ^27^, ^28^, ^45^, ^46^, ^47^, ^48^, ^51^, ^52^, ^53^, ^54^ Two studies reported predominantly Black participants and did not report White participants.^49^, ^50^ Hispanic or Latina/x/e participants were included in 11 studies,^15^, ^27^, ^28^, ^45^, ^46^, ^47^, ^48^, ^49^, ^50^, ^51^, ^54^ Asian participants were included in 5,^27^, ^46^, ^48^, ^51^, ^54^ and Native Hawaiian or Pacific Islander participants were included in one.^51^ Six studies reported participants who identified as multiracial or “other.”^15^, ^46^, ^47^, ^48^, ^51^, ^52^

Three studies explicitly enrolled participants who did not speak English: Lemon et al^28^ included English, Spanish, or Portuguese speakers, whereas Moustafa et al^47^ and Tully et al^54^ included English and Spanish speakers. Most other studies either restricted participation to English speakers or did not report language inclusion criteria, limiting assessment of linguistic representation and accessibility.

Reporting of sociodemographic variables was inconsistent. Insurance status was documented in all but one study.^53^ Geographic or neighborhood‐level data appeared in 6 studies,^28^, ^45^, ^47^, ^48^, ^53^, ^54^ income appeared in 3 studies,^50^, ^53^, ^54^ and education and employment appeared in only 2 studies.^53^, ^54^

Clinical characteristics were more consistently reported. Most studies described HDP severity and the use of oral antihypertensives at discharge. More than half documented comorbid conditions, such as gestational diabetes^15^, ^27^, ^28^, ^45^, ^46^, ^48^, ^49^, ^51^ or mental health conditions.^15^, ^28^ Mode of birth, gestational age, and parity were frequently included. Prenatal care use was reported in 3 studies,^15^, ^28^, ^51^ and only one study identified the type of postpartum care provider (ie, certified nurse‐midwife, general obstetrician‐gynecologist, maternal‐fetal medicine specialist, or “other”).^54^

### Assessment of BP Follow‐Up Across Studies

The included studies assessed postpartum BP follow‐up using various outcomes, including BP ascertainment (completion or documentation of a BP check), attendance at postpartum visits, and engagement in remote monitoring programs. Some studies measured ascertainment through documented BP values or completion of in‐office or remote BP evaluations.^27^, ^45^, ^50^, ^54^ Others evaluated attendance at postpartum BP visits.^15^, ^49^, ^52^ Several assessed enrollment or engagement in RBPM programs, such as submitting home BP readings or maintaining participation.^28^, ^46^, ^47^, ^48^ A few examined adherence to postpartum BP clinical practice guidelines^9^ or broader outcomes, such as BP control.^28^, ^48^, ^51^ One study explored perceptions of RBPM and nurse communication.^53^ These different outcome measures captured not only whether BP was obtained but also how results were communicated, acted on, and addressed by providers. Given this variability, the following results are organized around patient‐level, provider‐level, and system‐level factors influencing BP ascertainment and engagement, presented as barriers, facilitators, and predictors.

### Barriers

#### Race and Ethnicity

Multiple studies identified significantly lower postpartum BP ascertainment rates among non‐Hispanic Black patients compared with White patients.^27^, ^45^, ^49^, ^50^, ^52^ Two studies also reported lower enrollment and sustained engagement in RBPM among Black participants.^45^, ^47^ Moustafa et al found reduced participation among Hispanic patients even after adjusting for community‐level economic distress.^47^ Notably, Black and Hispanic patients were less likely to achieve BP ascertainment compared with non‐Hispanic White patients in similarly resourced communities, indicating inequities beyond socioeconomic status.^47^

#### Insurance and Economic Status

Insurance status, reflecting access to care, was associated with both postpartum BP ascertainment and engagement in RBPM. Lower ascertainment rates were consistently observed among patients with public insurance or no insurance.^28^, ^47^, ^48^, ^52^, ^53^ In contrast, commercial insurance and continuous Medicaid coverage were associated with higher RBPM participation.^28^ Community‐level indicators of economic hardship were also linked to reduced engagement.^47^ However, one study found no significant difference in BP ascertainment by socioeconomic status when comparing in‐person and telemedicine visits, suggesting that certain care models may help mitigate this barrier.^50^

#### Provider Communication and Bias

Several studies reported gaps in provider communication and patient education that affected both BP ascertainment and RBPM engagement. Moustafa et al found that limited prenatal counseling and a lack of individualized education reduced patient preparedness for postpartum BP monitoring.^47^ In the mixed‐methods study by Tully et al, participants described inpatient education as inadequate for recognizing or responding to abnormal BP, noting that handouts lacked depth and that discharge care was rushed, with frequent health care team members entering the room like a “revolving door.”^54^ Participants also reported uncertainty about maternal warning signs, with some unable to distinguish concerning symptoms from normal postpartum changes; one participant reported being unaware of the possibility of postpartum preeclampsia until after experiencing a hypertensive crisis.^54^ Another participant did not receive text messages because their phone line was inactive, highlighting how gaps in communication and technological access limited program engagement.^54^ Howard et al found that Black patients were less likely than White patients to enroll in a care management program designed to support postpartum BP ascertainment, even after accounting for health status and noted that enrollment was determined at provider discretion.^45^

#### Technology and System Barriers

Among patients enrolled in RBPM programs, several studies found that Black patients were less likely to enroll in or remain engaged.^28^, ^45^, ^47^, ^48^ Barriers included limited internet access, equipment issues, and competing life stressors.^45^, ^51^, ^53^ In Payakachat et al, participants also described difficulty incorporating twice‐daily monitoring into busy postpartum routines, stress related to abnormal readings, equipment bulkiness, and frequent BP reading transmission problems caused by poor cellular signal.^53^

#### Prenatal Care Gaps

Limited or fragmented prenatal care was also associated with decreased BP ascertainment and postpartum follow‐up.^15^, ^28^, ^49^, ^51^

### Facilitators

#### Remote Blood Pressure Monitoring

Most studies found RBPM improved postpartum BP ascertainment.^27^, ^28^, ^46^, ^47^, ^48^ For example, Arkerson et al reported that 92% of patients in the RBPM group completed a BP check within 10 days compared with 58% in‐office–based care, with racial disparities eliminated in the remote group.^27^ Participants also described valuing the convenience and reassurance of remote monitoring, which supported both program engagement and timely BP ascertainment.^54^ Text message reminders, especially when combined with structured support, were reported to be effective in sustaining engagement and improving the rate of follow‐up completion.^46^, ^51^, ^54^

#### Provider Communication and Education

Clear and supportive provider communication was frequently associated with higher BP ascertainment and engagement in monitoring programs.^27^, ^51^, ^52^ In the qualitative study, patients emphasized the importance of real‐time communication with nurses to interpret readings and guide responses.^53^ Patient education provided through nurse‐led interventions or QI bundles also facilitated timely BP ascertainment and follow‐up.^28^, ^51^

#### Language Inclusion

Multilingual access, such as translated educational materials or bilingual staff, facilitated both engagement in RBPM and timely BP ascertainment.^54^

#### Continuity of Care

Receiving prenatal care at the same hospital where birth occurred was associated with improved BP ascertainment, suggesting the benefits of integrated care distribution.^48^, ^51^

### Predictors

Several clinical and demographic characteristics functioned as predictors of postpartum BP ascertainment and follow‐up. Severe HDP, cesarean birth, and discharge on oral antihypertensives were consistently associated with higher BP ascertainment rates and early postpartum visit attendance.^15^, ^28^, ^49^, ^52^ Even so, disparities persisted: Black patients were less likely to achieve BP ascertainment despite having more severe HDP or being discharged on oral antihypertensives.^50^ Younger maternal age and multiparity were consistently associated with lower BP ascertainment and follow‐up,^15^, ^49^, ^50^, ^51^ whereas nulliparity was linked to higher rates.^52^

### Summary of Findings

Across the 13 studies, patient‐level, provider‐level, and system‐level factors shaped both early postpartum BP ascertainment and engagement in BP monitoring programs. Table 2 visually summarizes these barriers and facilitators, organized by level and direction of influence. Structural and social determinants, including racial and ethnic inequities, lack of or public insurance, economic disadvantage, fragmented prenatal care, and limited digital or in‐person access, posed major barriers to timely BP ascertainment. Provider‐level factors, such as communication gaps, inadequate patient education, and bias, influenced both BP ascertainment rates and patient engagement. Facilitators included RBPM, supportive communication, nurse‐led interventions, multilingual access, and continuity of care, all of which promoted higher BP ascertainment and sustained engagement. Clinical predictors, such as HDP severity, cesarean birth, and discharge on oral antihypertensives, were associated with higher BP ascertainment, whereas younger age and multiparity predicted lower rates. Despite improvements in BP ascertainment with RBPM and targeted interventions, racial and ethnic disparities—particularly among Black patients—remained persistent across studies.

**Table 2:** Multilevel Factors Influencing Early Postpartum BP Follow‐Up

Level	Barriers (−)	Facilitators (+)
Patient level	Young maternal age, multiparity, Black and Hispanic race/ethnicity, limited or no prenatal care, economic disadvantage, digital literacy gaps	Continuity of prenatal and postpartum care
Provider level	Limited prenatal counseling, inadequate or rushed discharge education, superficial patient handouts, provider discretion and potential bias in offering follow‐up programs	Clear and supportive provider communication, tailored patient education, real‐time nurse support, nurse‐led interventions, quality improvement bundles
System level	Structural racism, uninsured or publicly insured, limited prenatal care access, socioeconomic disadvantage, limited access to in‐person or telehealth follow‐up, technology barriers, digital access gaps	Remote BP monitoring, commercial or continuous Medicaid coverage, bilingual staff and translated materials, integrated prenatal and birth care at the same hospital, text message reminders, structured care management programs
Predictors^a^	HDP severity, discharge on oral antihypertensives, parity, cesarean birth	

Abbreviations: BP, blood pressure; HDP, hypertensive disorders of pregnancy.

a Predictors include nonmodifiable clinical and demographic factors associated with higher or lower follow‐up.

## DISCUSSION

This integrative review highlights persistent inequities in early postpartum BP follow‐up among individuals with HDP, particularly for Black and Hispanic postpartum persons and those experiencing socioeconomic disadvantage. Despite promising strategies (eg, RBPM and nurse‐led interventions), disparities in follow‐up were consistently observed, especially among Black persons. These patterns may reflect the influence of structural and social determinants of health on postpartum care engagement, including systemic racism, insurance gaps, and fragmented prenatal care, as summarized in Table 2. Addressing these inequities requires not only clinical innovation but systemic change to confront the structural conditions that perpetuate maternal health care disparities.

To better understand these disparities, this review synthesized findings through an intersectional lens attentive to how clinical, sociodemographic, and structural factors interact to influence postpartum BP follow‐up. Several demographic and clinical characteristics, such as race/ethnicity, maternal age, parity, HDP severity, and cesarean birth, emerged as predictors of follow‐up. Although they are not modifiable in the same way as structural conditions, these characteristics are deeply shaped by social contexts. For example, younger age and multiparity may reflect overlapping caregiving responsibilities or accumulated disadvantages that disproportionately affect Black and Hispanic persons and those with limited resources.^28^, ^56^

Notably, race and ethnicity were consistent predictors of lower completion of postpartum BP follow‐up, even after controlling for insurance status and clinical severity.^47^, ^50^ These disparities reflect how racism within health care systems shapes care experiences and opportunities for engagement, not race itself as a biological variable. In a 2022 qualitative study of the lived experiences of severe maternal morbidity in Black women, it was emphasized that it is racism—not race—that places women at risk within health care systems.^57^ Without explicitly addressing these structural forces, interventions risk reinforcing rather than reducing disparities.

Digital health care innovations, particularly RBPM, demonstrated improved BP ascertainment rates, yet significant access barriers persisted. Structural barriers to RBPM participation, such as limited broadband, lack of devices, and gaps in digital literacy, disproportionately affected Black and Hispanic individuals, rural residents, and those in economically distressed communities.^47^, ^51^, ^53^, ^54^ Beyond connectivity, participants in RBPM programs described difficulty integrating twice‐daily monitoring into busy postpartum routines, stress related to abnormal readings, and difficulties managing the equipment.^53^, ^54^ These findings illustrate how digital health care solutions can introduce new burdens without adequate support. From an intersectional perspective, these barriers demonstrate how race, geography, and socioeconomic position intersect to shape access to digital health care. Without intentional design, technological solutions may reproduce rather than reduce disparities. Programs that paired RBPM with culturally responsive education, technology support, and nurse facilitation achieved more equitable follow‐up,^27^, ^47^, ^54^ underscoring the importance of inclusive design centered on those most at risk of exclusion.

Provider communication emerged as a critical factor. Across studies, nurse‐led communication was associated with improved completion of general postpartum BP ascertainment and engagement in follow‐up care.^27^, ^51^, ^52^, ^54^ Participants in RBPM programs valued timely, supportive communication from providers.^53^, ^54^ These findings align with prior research in which women of color emphasized that person‐centered care, valuing lived experience, and respectful communication are essential to building trust and improving care experiences.^58^ However, even the best communication cannot fully overcome structural barriers. The interplay of race, language, and socioeconomic status, together with implicit bias and misalignment between provider communication and postpartum persons’ cultural or linguistic contexts, illustrates how intersecting factors shape whether care is received as respectful and trustworthy.^27^, ^45^, ^50^, ^54^ These findings are consistent with a recent integrative review by Goh et al, which highlights how communication gaps disproportionately affect pregnant people of color and contribute to inequitable care.^59^ Notably, the underrepresentation of non‐English speakers across most studies in this review limited the ability to fully assess how language access influences postpartum BP follow‐up. This represents a significant gap in the evidence base, as individuals with limited English proficiency face documented barriers to postpartum care and are underrepresented in maternal health research.

Limited or fragmented prenatal care, particularly when care was split across sites, was associated with decreased follow‐up, underscoring how gaps in continuity function as a structural barrier. Viewed through an intersectional lens, these predictors are not evenly distributed: severity and continuity of care often intersect with factors such as race, insurance status, and socioeconomic context, shaping who receives timely postpartum support. These findings suggest that structural access to care, rather than individual motivation, plays a critical role in follow‐up.

Looking forward, the midwifery model of care, with its emphasis on respectful, relationship‐centered communication,^60^, ^61^ may offer promise for equity‐focused postpartum BP surveillance. However, none of the included studies directly compared provider types, and access to midwifery care in the United States remains limited.^61^ Further research is needed to evaluate whether midwifery‐led care models improve BP follow‐up rates, particularly for women of color who face disproportionate barriers.

This review builds on prior literature by showing how clinical, sociodemographic, and structural factors converge to influence early postpartum BP follow‐up. Although systematic reviews demonstrate that RBPM improves timely BP follow‐up compared with in‐person care,^29^, ^31^ few studies have examined which populations remain excluded and why. This review addresses that gap by identifying how social and structural determinants of health care shape who benefits from RBPM and who continues to face barriers. Notably, several factors identified in this review (eg, race and ethnicity, parity, insurance coverage, and prenatal care use) mirror those linked to attendance at the 6‐week postpartum visit,^18^, ^62^, ^63^, ^64^ suggesting that barriers to early postpartum BP follow‐up reflect systemic shortcomings in maternal health care delivery across the postpartum continuum.

### Implications for Practice, Policy, and Research

Improving postpartum BP follow‐up requires equity‐centered strategies across practice, policy, and research. In clinical practice, interventions should directly address barriers identified in this review. Priorities include providing access to home BP monitors for early follow‐up after HDP, offering digital literacy support to facilitate RBPM participation, and ensuring flexible follow‐up options, such as RBPM, telehealth, or community‐based visits. Strengthening continuity of care—for example, by supporting follow‐up with the same clinician or care team who provided prenatal care—may also improve early postpartum follow‐up after HDP. Education should be individualized, linguistically accessible, and culturally responsive.

At the policy level, priorities include establishing reimbursement for RBPM and related supports, investing in digital infrastructure to address connectivity gaps, ensuring language access, and expanding community‐based, midwifery‐led care models that emphasize the continuity and communication quality identified as facilitators in this review.

Future research should adopt intersectionality as both a guiding framework and analytic tool, recognizing that it is not race itself but racialized experience within health care systems that drives inequities.^57^ Priorities include diversifying samples, using mixed‐methods and participatory designs, examining additional barriers (eg, substance use disorders, mental health conditions),^65^ and investigating structural determinants of care engagement. Qualitative studies are especially needed to capture the lived experiences behind the statistics, including how individuals perceive care, their understanding of HDP, and the barriers shaping follow‐up. Comparative effectiveness research should evaluate whether digitally enabled and collaborative care models involving midwives can address structural barriers and engage historically excluded populations.

Equity should not be an afterthought but the central objective of all maternal health care initiatives.

### Strengths and Limitations

This is the first integrative review to examine factors influencing completion of the 7‐to‐10‐day postpartum BP check among women of color with HDP using an intersectionality‐informed approach. Strengths include the inclusion of diverse study designs and participant populations, which enabled a nuanced synthesis of multilevel influences on postpartum care engagement. However, several limitations should be noted. The review process was conducted by a single reviewer, and most included studies did not explicitly apply intersectional frameworks or disaggregate findings by race, language, or other structural factors. Limited qualitative research and inconsistent reporting of sociodemographic variables across studies, particularly language inclusion, further constrained equity‐focused analysis. Despite these limitations, this review contributes important insights into the structural and systemic barriers to early postpartum BP follow‐up and supports the development of interventions, policies, and research that prioritize maternal health equity.

## CONCLUSION

This integrative review synthesized barriers, facilitators, and predictors of early postpartum BP follow‐up among women of color with HDP using an intersectionality‐informed lens. Findings demonstrate that structural forces, including racism, classism, inadequate insurance, digital exclusion, and limited access to quality care, shape follow‐up and continue to drive disparities. These inequities are not the result of individual behavior or preference but are rooted in sociopolitical systems that influence access, trust, and engagement in care.^66^ Achieving equitable early postpartum BP follow‐up will require coordinated strategies across clinical practice, policy, and research. Priorities include strengthening patient–provider communication, ensuring equitable access to RBPM and related supports, and evaluating the potential of midwifery and collaborative care models to address structural barriers. Clinicians must recognize the intersecting multilevel barriers identified in this review in order to tailor care that reduces disparities rather than reinforces them. Ultimately, improving outcomes for those at highest risk depends on sustained commitment to dismantling structural barriers and centering the voices and experiences of communities disproportionately affected by preventable complications and deaths related to HDP.

## CONFLICT OF INTEREST

The authors have no conflicts of interest to disclose.

## SUPPORTING INFORMATION

Additional supporting information may be found online in the online version of this article at the publisher's website.

**Appendix S1**. Preferred Reporting Items for Systematic Reviews and Meta‐Analyses (PRISMA) checklist.
